# The Fluctuation Trend of Serum Anti-SARS-CoV-2 IgM/IgG Antibodies Seroprevalence in the Non-COVID-19 Infected Population and Correlation with Peripheral Blood Leukocyte Parameters in Beijing, China, 2021: A Real-World Study

**DOI:** 10.3390/vaccines10040571

**Published:** 2022-04-07

**Authors:** Pan Wang, Nan Yang, Yuting Xue, Jiansuo Zhou, Yonghua Wu, Tiancheng Wang, Liyuan Cui

**Affiliations:** 1 Department of Laboratory Medicine, Peking University Third Hospital, Beijing 100191, China; wangpan@bjmu.edu.cn (P.W.); 2011210372@bjmu.edu.cn (Y.X.); zhoujiansuo@bjmu.edu.cn (J.Z.); yhwu@bjmu.edu.cn (Y.W.); 2 Core Unit of National Clinical Research Center for Laboratory Medicine, Beijing 100191, China; 3 Department of Blood Bank, Peking University Third Hospital, Beijing 100191, China; yangnan@bjmu.edu.cn

**Keywords:** COVID-19, SARS-CoV-2, vaccine, IgM and IgG antibody

## Abstract

Since 2019, the coronavirus disease 2019 (COVID-19) global pandemic has caused more than 300 million cases of disease and 5 million deaths. Vaccination has been widely accepted as the most effective measure for the prevention and control of this disease. However, there is little understanding about serum anti-SARS-CoV-2 IgM/IgG levels after inactivated vaccination as well as the relationship with peripheral blood leukocytes in the non-COVID-19 infected population. A total of 16,335 male and 22,302 female participants were recruited in this study, which was conducted in the Peking University Third Hospital located in Beijing (China). The level and seroprevalence of serum anti-SARS-CoV-2 receptor-binding domain (RBD) IgM/IgG and the association with peripheral blood leukocytes classification were investigated. With an increase in the number and percentage of full immunization of COVID-19 vaccinations in Beijing, serum anti-SARS-CoV-2 IgG antibodies levels and seroprevalence were significantly elevated (*p* < 0.01). The serum anti-SARS-CoV-2 IgG antibodies of 60 years and older persons were significantly lower than that of individuals that are 18~60 years old (*p* < 0.01), and there was a positive relationship between serum anti-SARS-CoV-2 IgG antibodies levels and peripheral blood lymphocyte count. The investigation of serum anti-SARS-CoV-2 IgM/IgG antibodies and the peripheral hematological index may prompt and help understand the adaptive immune response of vaccination.

## 1. Introduction

The 2019 coronavirus disease (COVID-19) caused by severe acute respiratory syndrome coronavirus 2 (SARS-CoV-2) has spread worldwide and had a detrimental impact globally, which presents seriously challenges to public health. Globally, the number of infected people has over 300 million and resulted in more than 5 million deaths as of 15 January 2022 (https://covid19.who.int, accessed on 27 March 2022). Until now, antibody-mediated immune responses to SARS-CoV-2 infection have been researched; the results show that antibodies play an essential role in COVID-19 disease development [1]. However, the adaptive immune responses after inactive vaccination in the real world are not clear.

Compared with other countries, China has been relatively successful in controlling COVID-19 outbreaks [2]. To control the virus effectively, vaccines might be the best measure to protect individuals and to prevent the spread [3,4]. Since January 2021, the COVID-19 vaccines have been authorized for use in Beijing residents of China. The cumulative administered dose and cumulative population of COVID-19 vaccination and the number and percentage of full immunization of COVID-19 vaccination from February to November of 2021 in Beijing grew rapidly. By November 2021, the cumulative administered dose and cumulative population of COVID-19 vaccination were up to 47.85 million doses and 21.14 million, respectively; by September 2021, the number and percentage of full immunization of COVID-19 vaccination among the permanent population aged 18 and above were up to 19.81 million and 98.49%, respectively (data from the Beijing municipal government and Beijing municipal health commission).

However, the relationship between COVID-19 vaccines with serum anti-SARS-CoV-2 IgM/IgG levels and peripheral blood leukocyte parameters is still not fully understood. In the present study, we aim to investigate the trend of serum anti-SARS-CoV-2 IgM/IgG levels and the hematological index during the period of COVID-19 vaccines to further evaluate the adaptive immune response to vaccination.

## 2. Materials and Methods

### 2.1. Study Population

Present clinical research was an observational, retrospective, real-world investigation. All laboratory measurements were performed at the Department of Laboratory Medicine of Peking University Third Hospital. Located in Beijing (China), the hospital has over 2000 clinical beds and is a modernized and comprehensive upper first-class hospital with an integration of medical services, education, scientific research, disease prevention, rehabilitation, and health care. The data were routinely recorded in the database of clinical laboratory information systems and hospital information systems. The data of the present study were extracted from the database above. As a real-world study, the investigation did not design any inclusion or exclusion criteria, apart from the exclusion of patients with severe chronic diseases. A total of 38,637 subjects (16,335 male and 22,307 female) aged 0~101 years old were recruited, and those subjects underwent health check-ups at the outpatient department of the hospital from February to November 2021. The nasal or mouth throat swab specimens for SARS-CoV-2 RNA determination and the blood samples for peripheral blood leukocyte analysis and anti-SARS-CoV-2 IgM/IgG antibodies analysis were collected at the same time, and the testing was completed in the next few hours. This study was approved by the Medical Science Research Ethics Committee of Peking University Third Hospital (No. 2022-009-01).

### 2.2. Sample Collection and Processing

#### 2.2.1. Nasal or Mouth Throat Swab Specimen Collection

Mouth throat swab: Use a plastic rod swab with a polypropylene fiber tip to wipe both pharyngeal tonsils and posterior wall of the pharynx at the same time; immerse the tip of the swab in a tube of 3 mL sampling solution; discard the tail; and tighten the cap. Nasal throat swab specimen: Use a plastic rod swab with a polypropylene fiber head and insert it into the nasal passage and the nasal palate for a while, and then slowly turn to exit. The swab is immersed in a tube of 3 mL sampling solution, the tail is discarded, and the tube cap is tightened.

#### 2.2.2. The Peripheral Blood Sample Collection

The blood sample was obtained by venipuncture and collected into vacuum blood collection tubes (INSEPACK^®^, Sekisui, Beijing, China). The peripheral whole blood samples were collected into tubes containing EDTA-K^2+^ for peripheral blood leukocyte analysis or the procoagulant inert separation tube for the analysis of serum anti-SARS-CoV-2 IgM/IgG antibodies. Then, the serum samples in the procoagulant inert separation tube were obtained by centrifugation for 10 min at 3500 rpm to precipitate the cellular components of the blood specimen.

### 2.3. Analytical Procedures and Instruments

#### 2.3.1. SARS-CoV-2 RNA Determination

SARS-CoV-2 nucleic acid was detected with the real-time fluorescence PCR (RT-PCR) method. RT-PCR method used the SARS-CoV-2 ORF Lab and N gene to design specific primers and TaqMan probes for amplification and detection with a fluorescent quantitative PCR instrument. In this study, the SARS-CoV-2 nucleic acid in the sample was measured with the kit produced by Shanghai ZJ Bio-Tech Co., Ltd., Shanghai, China, and was carried out following the kit’s manual instructions; the Thermo Fisher ABI7500 PCR amplification instrument was used for amplification detection [5].

#### 2.3.2. The Semi-Quantitative Analysis of Serum SARS-CoV-2 IgM/IgG Antibodies

The serum SARS-CoV-2 IgM/IgG antibodies were measured with the magnetic particle chemiluminescence semi-quantitative method by using the AutoLumo A2000 Plus fully automated chemiluminescence Immunoassay analyzer and supporting reagents kits (Autobio Diagnostics Co., Ltd., Zhengzhou, Henan Province of China) [6]. This assay measured the IgM/IgG antibody designed to detect specific IgM/IgG antibodies to the receptor-binding domain (RBD) of the S1 subunit of SARS-CoV-2 spike protein in human serum or plasma. The measuring principle is as follows: anti-human IgM/IgG antibody was used to coat the magnetic particles, and the SARS-CoV-2 antigen (recombinant SARS-CoV-2 RBD fragment) was labeled with horseradish peroxidase to prepare the enzyme conjugate. During the immune reaction, a solid-phase secondary antibody of IgM/IgG antibody-enzyme-labeled antigen complex was formed, which can further catalyze the emission of photons from the luminescent substrate, and the luminescence intensity is proportional to the level of anti-SARS-CoV-2 IgM/IgG antibody in the sample. When the measured value of the signal to the cut-off ratio (S/CO) was greater than or equal to 1, the result was judged as positive; when the measured value is less than 1, the result is judged as negative.

#### 2.3.3. The Peripheral Blood Leukocyte Analysis

The peripheral leukocyte parameters were analyzed within 2 h after blood sample collection. The peripheral white blood cell (WBC) parameters including leukocyte count and differential count including the neutrophils, lymphocytes, eosinophils, basophils, and monocytes were determined with the XN hematology auto-analyzer (SYSMAX Inc. Kobe, Japan).

### 2.4. Statistical Analysis

The Statistical Package of Social Science (SPSS) 25.0 (SPSS Inc., Chicago, IL, USA) was used for statistical analysis. Data visualization was performed with the packages of corrplot (version 0.84) and ggplot2 (version 3.1.1) of R (version 3.6.1) (https://cran.r-project.org/bin/windows/base/, accessed on 27 March 2022) and RStudio (version 1.2.1335) (version 1.2.1335, https://www.rstudio.com/, accessed on 27 March 2022). The one-sample Kolmogorov–Smirnov method to assess the type of data distributions. The χ^2^ test was used to compare differences in enumeration data. For bivariate analyses, Spearman’s correlation was performed. The serum antibody between different groups was performed with a Mann–Whitney U nonparametric test. Spearman’s correlation was performed for bivariate analyses. Multivariate analysis was performed with generalized linear mixed models (GLMMs). For all tests, the *p* values reported were two-tailed and considered statistically significant when *p* < 0.05.

## 3. Results

### 3.1. The Distribution, Levels, and Monthly Positive Percentage of Serum Anti-SARS-CoV-2 IgM/IgG and Peripheral Blood Leukocyte Analysis in the Study Population

**Figure 1:**
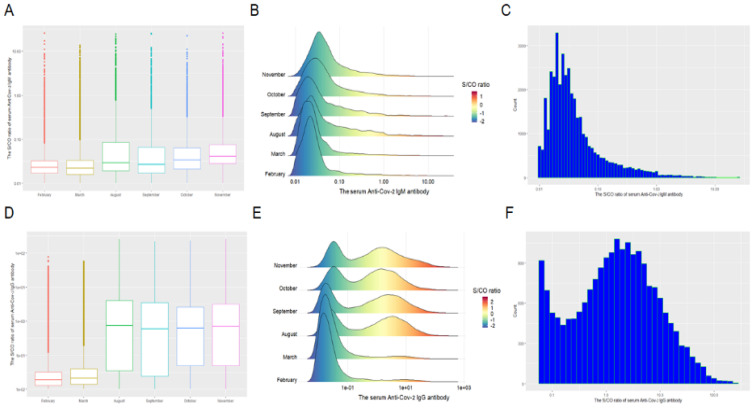
The average of S/CO ratio (**A**,**D**), S/CO ratio density (**B**,**E**), and distribution (**C**,**F**) of serum anti-SARSCoV-2 IgM/IgG antibodies of the present study population in February, March, August, September, October, and November in Beijing.

**Figure 2:**
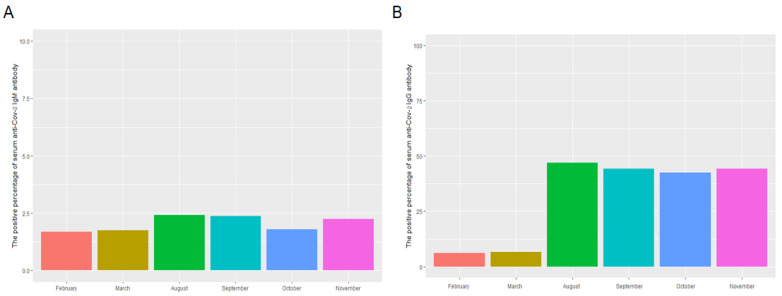
The positive percentage of serum anti-SARS-CoV-2 IgM/IgG antibodies of the present study population in February, March, August, September, October, and November in Beijing (**A**,**B**).

The distribution, levels, and monthly positive percentage of serum anti-SARS-CoV-2 IgM and IgG as well as the result of peripheral blood leukocytes in the study population are shown in Table 1 and Figure 1 and Figure 2. The median of S/CO and positive percentage of serum anti-SARS-CoV-2 IgM antibody on February, March, August, September, October, and November 2021 were 0.023 and 1.68%; 0.022 and 1.74%; 0.029 and 2.42%; 0.027 and 2.38%; 0.034 and 1.80%; and 0.041 and 2.25% respectively (Figure 1A–C). The median of S/CO and positive percentage of serum anti-SARS-CoV-2 IgG antibody on February, March, August, September, October, and November 2021 were 0.019 and 6.17%; 0.021 and 6.57%; 0.746 and 47.01%; 0.599 and 44.27%; 0.623 and 42.45%; and 0.710 and 44.29%, respectively (Figure 1D–F). The total positive percentages of serum SARS-CoV-2 IgM and IgG were 2.04% and 31.79%, respectively. The levels and monthly positive percentage of serum anti-SARS-CoV-2 IgM and IgG in August, September, October, and November were significantly higher than that in February and March (Figure 2A,B).

### 3.2. The Levels of Serum SARS-CoV-2 IgM and IgG in the Different Age Group

**Figure 3:**
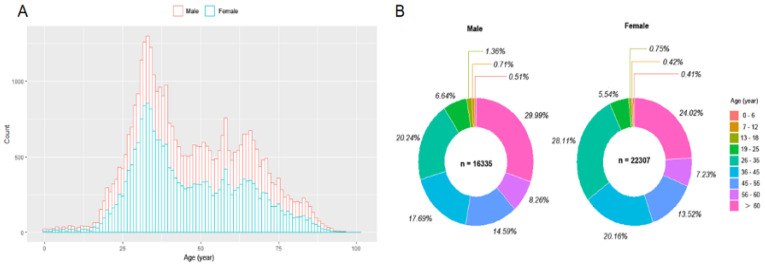
The age distribution and composition of the present study population (**A**,**B**).

**Figure 4:**
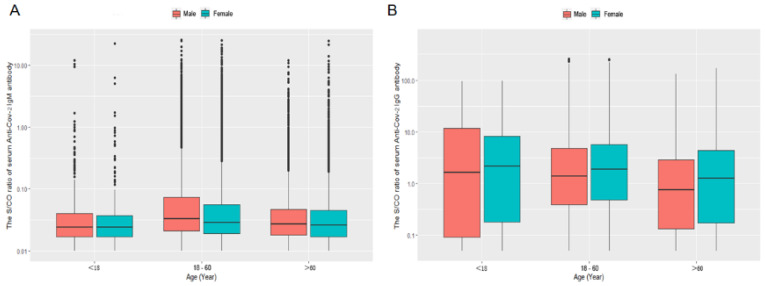
The average S/CO ratio of serum anti-SARS-CoV-2 IgM/IgG antibodies in the age groups of <18, 18~60, and >60 years old (**A**,**B**).

The age constitution of the present study population was shown Figure 3A,B. The percentages of age constitution on less than 18, 18~60, and over 60 years old were about 1.61%, 71.85%, and 26.54% in present study subjects. Both in male and female, the serum anti-SARS-CoV-2 IgM levels of 18~60 years old group were significantly higher than that of the <18 and >60 years old group (*p* < 0.001 and < 0.001, respectively) but without being significantly different from that of the >60 years old group (*p* = 0.067 and 0.149, respectively). The male’s serum anti-SARS-CoV-2 IgM levels of 18~60 and >60 years old were significantly higher than that of females in the same age group (*p* < 0.001 and 0.006, respectively). There was no significant difference in serum anti-SARS-CoV-2 IgM levels between males and females in the less than 18 years old group (*p* = 0.735) (Figure 4A).

Both in the male and female, the serum anti-SARS-CoV-2 IgG levels of <18 years old group were significantly high than that of the 18~60 and >60 years old group (*p* < 0.001 and < 0.001, respectively), and the serum anti-SARS-CoV-2 IgG levels of 18~60 were higher than >60 years old group (*p* < 0.001). The male’s serum anti-SARS-CoV-2 IgG levels of 18~60 and >60 years old were significantly lower than that of females in the same age group (*p* = 0.005 and 0.022, respectively). There was no significant difference in serum anti-SARS-CoV-2 IgG levels between males and females in the less than 18 years old group (*p* = 0.658) (Figure 4B).

### 3.3. The Correlation between the Serum Anti-SARS-CoV-2 IgM and IgG Antibodies

**Figure 5:**
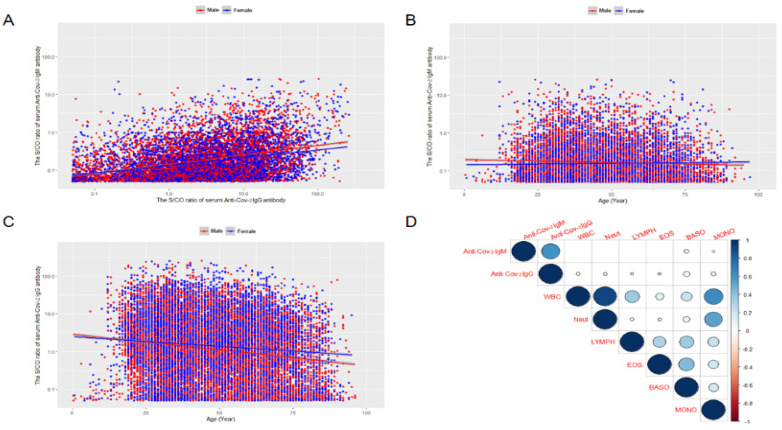
The relationship between the S/CO ratio of serum anti-SARS-CoV-2 IgM/ and IgG in the present study population (**A**). The association between the age and the S/CO ratio of serum anti-SARS-CoV-2 IgM/IgG in the present study population (**B**,**C**). Spearman’s correlation between the S/CO ratio of serum anti-SARS-CoV-2 IgM/IgG and peripheral blood white blood cell classification (**D**).

The result of the spearman correlation analysis showed that the levels of serum anti-SARS-CoV-2 IgM and IgG antibodies were significant (*p* < 0.001) in both males and females (Figure 5A).

### 3.4. The Relationship between the Serum Anti-SARS-CoV-2 IgM/IgG Antibodies Levels and Age

There was a significant correlation between the age and serum anti-SARS-CoV-2 IgM antibody levels in both males and females (*r* = −0.118, and −0.047, respectively, both the *p* < 0.001) (Figure 5B). In addition, age and serum anti-SARS-CoV-2 IgG antibody levels also had a significant correlation (*r* = −0.120 and −0.027, respectively, both the *p* < 0.001).

### 3.5. The Relationship Serum Anti-SARS-CoV-2 IgM/IgG Antibodies Levels and Peripheral Blood Neutrophils or Lymphocytes by Generalized Linear Mixed Model (GLMMs) Analysis

GLMMs were used to analyze the relationship between serum anti-SARS-CoV-2 IgM/IgG antibodies levels and age by controlling important confounding factors including sex and time of the study subjects (Table 2 and Figure 5D). Only the fixed effect in GLMMs was observed. The results showed that the serum anti-SARS-CoV-2 IgG antibody had a significantly negative correlation with peripheral blood neutrophils and positively correlated with peripheral blood lymphocytes after adjusting for other confounding factors (*p* < 0.001).

## 4. Discussion

Vaccines have been widely regarded as the best method to control SARS-Cov-2 infection [7]. In Beijing, residents aging from 18 to 60 years old successively started to get vaccinated with inactivated SARS-CoV-2 vaccine developed by Beijing Sinovac Science & Technology Co., Beijing, China, LTD, or inactivated SARS-CoV-2 vaccine (Vero cells) from the Beijing Institute of Biological Products/Sinopharm, Beijing, China in February 2021; 2 weeks after the first vaccination, the booster shot was provided. In October 2021, the third vaccine was provided again.

Serum immunological testing is a common method for detecting COVID-19 [8,9,10]. The common method for assessing the effectiveness of COVID-19 vaccines is to measure the level of neutralizing antibodies in the blood. Serum anti-SARS-CoV-2 RBD IgM/IgG antibodies are part of neutralizing antibodies [11]. Another study observed a strong correlation between levels of RBD binding antibodies and SARS-CoV-2 neutralizing antibodies in patients [12]. The measurement of anti-SARS-CoV-2 RBD IgG antibody response is very important in order to define the dynamics of immunization in vaccine COVID-19 recipients. Malipiero et al. used a commercial anti-RBD (receptor binding domain) IgG quantitative chemiluminescent immunoassay to investigate the kinetics of anti-SARS-CoV-2 neutralizing antibodies on Pfizer/BioNTech BNT162b2 vaccine in local healthcare workers [13,14]. Another study suggested that the antibody’s value of the anti-RBD IgG antibody might reflect the anti-SARS-CoV-2 neutralizing bioactivity to some extent in the body’s immune response to COVID-19 inactive vaccination and has a certain application value to explore the immune effect of the vaccinated population for the prevention of COVID-19 infections [15].

Thus far, several reports analyzed serum anti-SARS-CoV-2 IgM/IgG antibody seroprevalence among the non-COVID-19 infected population or COVID-19 infected population [16,17,18,19,20]. To the best of our knowledge, there have been no reports concerning the large scale real-world serum anti-SARS-CoV-2 IgM/IgG antibodies levels and the hematological index and its relationship with vaccination in the non-COVID-19 infection population. The present study showed that with the cumulative increase in the population of COVID-19 vaccination, the median of S/CO and positive percentage of serum anti-SARS-CoV-2 IgG antibody from August to November of 2021 was higher than that of February and March in Beijing, China. Therefore, we confirm that the SARS-CoV-2 vaccine can activate the body’s immune response. Our study manifested that 47.01% of the resident population in the present study had a significant IgG antibody response to inactivated SARS-CoV-2 vaccine in August 2021, which was slightly lower than that of the previous study, which showed that 74.2% of the participants who received the second dose of the vaccine had a positive IgG antibody response [21]. The reasons may be that although the percentage of COVID-19 vaccine with full immunization in Beijing exceeded 90% in August 2021, and a portion of the recruited population includes migrant patients. Moreover, vaccination for subjects aged from 6 months to 18 years old (about 2% in the present investigation population) will not be in progress until the end of October 2021. Secondly, this study is a time-series trend study. The vaccination rate in Beijing from August to November in 2021 is significantly higher than that in February and March. However, in a specific month, the age composition of the population is different, and the vaccination situation is also different. Thirdly, previous studies on the immune response to SARS-COV-2 vaccine are prospective cohort studies of small populations, which can specifically observe antibody level in serum after the first, second, and booster doses of SARS-COV-2 vaccine. However, this study is a time-series large population study, and our study focuses on the macroscopic relationship between the overall serum antibodies level and positive rate with time; thus, the results of the study may not be consistent with other studies. Finally, Serum SARS-COV-2 IgG antibody kits used in his study are different from others used in the previous reports, and the results of the study are closely related to the cutoff values set by different manufacturers because the reagent manufacturers need to comprehensively consider the specificity and sensitivity of the detection method to set cutoff values [18,22]. Therefore, our results reflected the actual seropositivity level of IgG and the status of herd immunity of the resident population in that period.

The total seropositivity rate of serum IgM and IgG antibodies in August in the present study population was higher than that in February and March, and the serum IgG and IgM antibody levels gradually decreased over time in September and October. This decline suggested that the IgG antibody could not maintain at a higher level for a longer time in the body, and it is necessary to strengthen vaccination [6,23]. After beginning the booster vaccination at the end of October in Beijing, the IgG antibody seropositivity rate in November increased compared with that in September and October. This proves once again that the booster vaccine can enhance the human immune system to produce anti-SARS-CoV-2 RBD IgG antibodies.

The bivariate Spearman correlation showed that serum SARS-CoV-2 antibody levels were negatively correlated with peripheral blood white blood cell counts and classification. However, Spearman’s correlation analysis can only reveal the preliminary relationship between the bivariate, and it is difficult to rule out the influence of confounding factors. Considering the complex impact of age, gender, and time on serum ARS-CoV-2 RBD IgM/IgG antibodies, we performed GLMMs analysis, which has the advantage of controlling the important confounding factors of study subjects and can deal with non-normally distributed data. GLMMs analysis showed that the serum anti-SARS-CoV-2 IgM/IgG antibody level did not have a significant difference between gender, but it was negatively correlated with age. The mean serum anti-SARS-CoV-2 IgG antibody level of the older groups (>60 years old) was lower than that of the younger group, and the anti-SARS-CoV-2 IgM/IgG antibody showed a gradual downward trend related to increased age. Our finding is consistent with earlier studies that older persons had lower levels of antibodies than young subjects [24]. This implied that the elderly population had a decreased human immune response to COVID-19 inactive vaccination and immune senescence may be the cause of this phenomenon, the COVID-19 inactive vaccine is less protective in older people than in younger ones.

Previous studies demonstrated that patients infected with COVID-19 generally presented lymphocytopenia and neutrophilia [25,26,27]. On the other hand, GLMMs analysis in this study showed that the serum anti-SARS-CoV-2 IgG antibody level was positively associated with the number of peripheral blood lymphocytes and negatively associated with the amount of peripheral blood neutrophil counts after adjusting for age, gender, and time covariables. It suggested that there are some differences in peripheral blood leukocytes between patients with COVID-19 infection and vaccine. The reason may be due to the difference in mechanisms in immune response induced by viral infection and vaccination [28,29]. When COVID-19 induces viral infections, the B cells induced by follicular helper CD4^+^ T cells could produce antibodies capable of neutralizing the pathogen and can combine with cytolytic CD8^+^ T cells to kill pathogen-infected cells, which leads to a decrease in peripheral blood lymphocytes, while the COVID-19 vaccine could induce robust lymphocyte generation, which led to elevated peripheral blood lymphocytes.

Our study demonstrated several strengths. First, it is a real-world approach and included the largest cohort of serum anti-SARS-CoV-2 IgM/IgG antibodies levels and the relationship with vaccination in non-COVID-19-infected population ever evaluated in the literature, which reflected objectively the real status of the serum anti-SARS-CoV-2 IgM/IgG antibody. Second, the relationship between serum anti-SARS-CoV-2 IgM/IgG antibody level and peripheral blood leukocytes was investigated. However, there are still several limitations that should be underlined. Firstly, the present study was a retrospective study performed with real-world data, which lacks detailed information on an individual’s vaccination time. Thus, the methodological limits and the high potential heterogeneity as well as possible confounding factors, apart from age, among the subjects included should be considered. Secondly, there was no actual overall serum anti-SARS-CoV-2 neutralization antibody level and of the study population during the vaccine, which makes it hard to accurately assess the response process and situation of immunity during vaccine with inactivated SARS-CoV-2. Future studies on this topic should consider the specific vaccine time, including the first, second, and third vaccine booster dose. Thirdly, although the results of the present study suggest that the serum anti-SARS-CoV-2 IgM/IgG antibodies levels elevated when the individual obtained the inactivated SARS-CoV-2 vaccine, the protective effect of the vaccine was assessed indirectly by detecting serum antibody levels when only providing a limited reference for immune status in the resident population. A more intensive and comprehensive investigation with more populations and regions as well as observation time is needed to verify the results of the presents study.

## 5. Conclusions

With an increase in the number and percentage of full immunization of COVID-19 vaccination, the serum anti-SARS-CoV-2 IgG antibodies levels and seroprevalence of the resident population were significantly elevated from February to November of 2021 in Beijing. The serum anti-SARS-CoV-2 IgG antibodies in 60 years and older persons were significantly lower than that of those that are 18~60 years old. Our present real-world study was designed to evaluate the serum anti-SARS-CoV-2 IgM/IgG antibodies levels and fluctuation trend in the non-COVID-19 infection population. This study will provide beneficial help for public health policy decisions such as the immunity assessment on COVID-19 and the suitable time interval of vaccination boosters as well as understanding the machine of immunologic effects on the body after the SARS-CoV-2 vaccine.

## Figures and Tables

**Table 1:** Characteristics of the study population.

		Male (*n* = 16,335)	Female (*n* = 22,302)	ALL (*n* = 38,637)
	Unit	Mean ± SD/Median (Range)		
Age	Year	48.71 ± 18.45/48.00 (98.00)	46.16 ± 17.24/42.00 (101.00)	47.24 ± 17.81/44.00 (101.00)
SARS-CoV-2 nucleic acid		Negative		
Serum Anti-SARS-CoV-2 antibody				
IgM	S/CO	0.16 ± 0.75/0.03 (25.78)	0.13 ± 0.72/0.03 (25.29)	0.14 ± 0.73/0.03 (25.78)
IgG	S/CO	3.00 ± 10.82/0.08 (255.77)	3.20 ± 10.89/0.06 (254.10)	3.11 ± 10.86/0.07 (255.77)
The parameters of peripheral blood leukocytes				
Leukocyte	(×10^9^/L)	7.06 ± 2.37/6.67 (67.93)	6.86 ± 2.36/6.45 (42.45)	6.95 ± 2.37/6.55 (68.37)
Neutrophils	(×10^9^/L)	4.44 ± 2.19/3.99 (65.56)	4.42 ± 2.19/3.94 (41.72)	4.43 ± 2.18/3.96 (65.57)
	%	61.28 ± 10.74/60.70 (92.20)	62.58 ± 10.73/62.20 (91.95)	62.03 ± 10.76/61.60 (93.00)
Eosinophils	(×10^9^/L)	0.15 ± 0.16/0.11 (6.03)	0.11 ± 0.19/0.08 (21.16)	0.13 ± 0.18/0.90 (21.16)
	%	2.20 ± 2.09/1.70 (46.00)	1.68 ± 1.82/1.20 (73.20)	1.90 ± 1.96/1.40 (73.20)
Basophils	(×10^9^/L)	0.04 ± 0.02/0.03 (0.54)	0.03 ± 0.02/0.03 (0.65)	0.03 ± 0.02/0.03 (0.65)
	%	0.54 ± 0.31/0.50 (7.60)	0.49 ± 0.29/0.50 (7.20)	0.52 ± 0.30/0.50 (7.60)
Lymphocytes	(×10^9^/L)	2.00 ± 0.75/1.95 (12.87)	1.92 ± 0.67/1.87 (15.91)	1.95 ± 0.71/1.90 (15.91)
	%	29.68 ± 9.96/30.10 (87.00)	29.60 ± 9.82/29.80 (88.90)	29.63 ± 9.88/29.90 (89.00)
Monocytes	(×10^9^/L)	0.43 ± 0.16/0.40 (3.49)	0.38 ± 0.15/0.35 (10.95)	0.40 ± 0.16/0.37 (10.95)
	%	6.29 ± 1.88/6.00 (34.50)	5.64 ± 1.69/5.40 (42.60)	5.92 ± 1.81/5.70 (42.60)

S/CO: Signal to cut-off ratio.

**Table 2:** GLMMs analysis on the relationship between serum anti-SARS-CoV-2 IgM/IgG antibody and the parameters of peripheral blood leukocytes in the study subjects adjusted by time (day).

Model Term		Coefficient	Std. Error	*t*	*p*	95% CI
IgM	Intercept	−4.119	0.669	−6.161	<0.001	−5.430–−2.809
	Age	−0.001	0.000	−4.181	<0.001	−0.001–−0.000
	Sex	0.036	0.008	4.637	<0.001	0.021–0.051
	Neutrophils	−0.005	0.002	−2.433	0.015	−0.009–−0.001
	Eosinophils	0.012	0.022	1.426	0.583	−0.031–0.056
	Basophils	0.059	0.199	0.477	0.634	−0.296–0.486
	Lymphocytes	0.008	0.006	1.426	0.154	−0.003–0.020
	Monocytes	−0.049	0.029	−1.698	0.090	−0.105–0.008
IgG	Intercept	−312.912	9.782	−31.99	<0.001	−332.084–−293.739
	Age	-0.038	0.003	−12.29	<0.001	−0.045–−0.032
	Sex	0.008	0.113	0.067	0.947	−0.214–0.230
	Neutrophils	−0.103	0.030	−3.459	0.001	−0.162–−0.045
	Eosinophils	−0.149	0.325	−0.459	0.647	−0.787–0.488
	Basophils	−1.956	2.918	−0.670	0.503	−7.676–3.764
	Lymphocytes	0.274	0.086	3.179	0.001	0.105–0.443
	Monocytes	−0.660	0.422	−1.564	0.118	−1.487–0.167

CI: Confidence Interval.

## Data Availability

The data supporting the finding of this study are available upon reasonable request from the authors.
